# Enhancing salinity tolerance in cucumber through Selenium biofortification and grafting

**DOI:** 10.1186/s12870-023-04711-z

**Published:** 2024-01-03

**Authors:** Masoomeh Amerian, Amir Palangi, Gholamreza Gohari, Georgia Ntatsi

**Affiliations:** 1 Department of Horticultural Sciences and Engineering, Faculty of Agricultural Sciences and Engineering, Campus of Agriculture and Natural Resources, Razi University, Kermanshah, Iran; 2 Department of Horticultural Sciecne, Faculty of Agriculture, University of Maragheh, Maragheh, Iran; 3 Department of Crop Science, Laboratory of Vegetable Crops, Agricultural University of Athens, Athens, Greece

**Keywords:** Antioxidant enzymes, Compatible solutes, Pepo, Rootstock

## Abstract

**Background:**

Salinity stress is a major limiting factor for plant growth, particularly in arid and semi-arid environments. To mitigate the detrimental effects of salinity stress on vegetable production, selenium (Se) biofortification and grafting onto tolerant rootstocks have emerged as effective and sustainable cultivation practices. This study aimed to investigate the combined effects of Se biofortification and grafting onto tolerant rootstock on the yield of cucumber grown under salinity stress greenhouse conditions. The experiment followed a completely randomized factorial design with three factors: salinity level (0, 50, and 100 mM of NaCl), foliar Se application (0, 5, and 10 mg L^-1^ of sodium selenate) and grafting (grafted and non-grafted plants) using pumpkin (*Cucurbita maxima*) as the rootstock. Each treatment was triplicated.

**Results:**

The results of this study showed that Se biofortification and grafting significantly enhanced salinity tolerance in grafted cucumbers, leading to increased yield and growth. Moreover, under salinity stress conditions, Se-Biofortified plants exhibited increased leaf relative water content (RWC), proline, total soluble sugars, protein, phenol, flavonoids, and antioxidant enzymes. These findings indicate that Se contributes to the stabilization of cucumber cell membrane and the reduction of ion leakage by promoting the synthesis of protective compounds and enhancing antioxidant enzyme activity. Moreover, grafting onto pumpkin resulted in increased salinity tolerance of cucumber through reduced Na uptake and translocation to the scion.

**Conclusion:**

In conclusion, the results highlight the effectiveness of Se biofortification and grafting onto pumpkin in improving cucumber salinity tolerance. A sodium selenate concentration of 10 mg L^-1^ is suggested to enhance the salinity tolerance of grafted cucumbers. These findings provide valuable insights for the development of sustainable cultivation practices to mitigate the adverse impact of salinity stress on cucumber production in challenging environments.

## Background

Salinity stress is widely recognized as a major constraint for agricultural productivity in tropical and subtropical regions. It leads to a decrease in crop productivity and product quality, thereby posing significant challenges to global food security [1]. Approximately 20% of arable land worldwide is severely damaged by salinity, with the remaining half being affected to varying degrees. There are two forms of soil saline process. Primary or natural salinity and secondary or human salinity. Various hydrological, geomorphic and climatic factors are the primary causes of saline soil. Secondary soil salinity is caused by low water levels, poor water irrigation, over irrigation with inadequate drainage, overuse of ground-water in coastal areas and unsolvable industrial wastewater, and Sewage with high soluble salt. In particular, secondary salinization can stem from different human driven processes [2]. High salinity levels in the soil can result in osmotic balance disruption, limiting water intake and transpiration and consequently yield [3]. The impact of salinity stress on plant growth is complex and depends on several factors, including the level of salinity, type of salt, and the specific plant species involved [4].

Traditional breeding programs have been extensively utilized to enhance crop salinity tolerance. However, achieving commercial success has proven difficult, primarily due to the complexity of this phenomenon. The genetic and physiological traits associated with tolerance to environmental stresses, including salinity, pose significant obstacles in conventional breeding approaches [5]. To address these challenges, gene transfer methods are currently employed to enhance tolerance to salt, although achieving tolerance to multiple stresses through gene transfer is difficult. Recently, grafting onto tolerant rootstocks has emerged as a promising and environmentally friendly technique for enhancing crop yield, offering benefits such as resistant to pests, diseases, and environmental stress [6, 7, 8, 9, 10, 11 and 12].

One of the environmentally friendly ways to mitigate yield reduction and increase resistance to soil diseases (especially damping off) and environmental stresses in the Solanaceae and Cucurbitaceae genotypes is to graft them onto resistant rootstocks [8, 13]. This technique allows plant breeders to harness the advantageous traits of both the rootstock and scion. Rootstock can significantly affect plant growth, yield, and fruit quality [7, 14, 15 and 16]. Numerous reports emphasize the pivot role of rootstock selection in conferring tolerance to environmental stresses, pathogens, and suboptimal soil growth conditions. Successful grafting ensures the scion’s ability to yield high and provide products of superior quality, while the rootstock increases stress tolerance related to the soil conditions. The interaction between rootstock and scion plays a crucial role in determining scion’s tolerance to environmental stresses [17]. In grafted plants, tolerance to salinity stress can be attributed to the accumulation of proline and total soluble sugars [18], enhanced antioxidant capacity [19], and reduced of sodium and chlorine accumulation in the scion [20].

Grafting is a reciprocal process, in which both the rootstock and the scion affect plant’s tolerance to salinity stress [21]. Studies in potato (*Solanum tuberosum* L.) [22], melon (*Cucumis melo* L.) [23], tomato (*Solanum lycopersicum* L.) [24], watermelon (*Citrullus lanatus* L.) [25], and cucumber (*Cucumis sativus* L.) [26, 27] plants highlighted the importance of rootstock in conferring salinity stress tolerance in grafted plants. In tomato, however, both the rootstock and scion contribute to salinity stress tolerance [28].

Cucumber is low-calorie vegetable rich in minerals and phenolic compounds. As a glycophyte plant, cucumber is extremely sensitive to soil salinity. Salinity stress adversely affects cucumber growth as a result of osmotic stress, which is followed by ion toxicity. The osmotic stress leads to nutrient imbalances, reactive oxygen species (ROS) production, and membrane damage thereby reducing yield and product quality [29]. Research indicates that high salinity tolerance in grafted cucumber plants is linked to increased leaf potassium concentration [20]. Grafting cucumber onto fig leaf gourd (*Cucurbita ficifolia* Bouche L.) has shown to increase yield and tolerance to salinity [29, 30]. However, cucumber fruit quality and taste can be negatively affected necessitating a careful rootstock selection to increase tolerance both under abiotic and biotic stresses, while improving the yield and quality of grafted cucumber fruit. Luffa (*Luffa aegyptiaca* L.) has been introduced as a promising rootstock for cucumber, demonstrating increased salt-resistant and cucumber growth. According to Guo et al. [31], this growth increase can be attributed to increased plant height, leaf number, photosynthesis, antioxidant activity, total soluble sugars, and potassium accumulation in aerial plant parts.

Selenium is an essential micronutrient crucial for animal and human health as well as plant growth and development [32]. To improve the quality of agricultural products and mitigate Se deficiency problems in society, the biological addition of Se, widely known as Se biofortification, has gained attention [33]. The World Health Organization (WHO) has recommended a daily intake of approximately 55 µg Se for adults. Selenium deficiency directly affects human health since more than 40 types of diseases, such as Keshan 2 disease, cancer, cardiovascular diseases, liver diseases, and cataracts, have been linked to its inadequate levels in the human body. Plants play a crucial role in transferring Se from the soil to the human food chain [34].

In plants, Se has emerged as a beneficial element that can mitigate the adverse effects of heat stress [35], heavy metals [36], ultraviolet radiation [37], drought [38], and salinity [39]. According to Lan et al. [40], Se can help alleviate the oxidative damaged induced by stress due to the increased activity of antioxidant enzymes (peroxidase, catalase, etc.) and number of antioxidant compounds in the body (anthocyanins, flavonoids, phenolic compounds, etc.). Selenium is one of the essential components of the antioxidant enzyme system aiding in the scavenging of free radicals produced by salinity stress conditions [40]. This leads to improved photosynthesis, ion homeostasis and increased plant growth and yield [41]. Moreover, appropriate levels of Se have been shown to reduce the negative effects of salinity stress by enhancing the plant’s defense mechanism and regulating sodium carriers [42]. As shown by Regni et al. [43], Se increased tolerance to salinity stress in olive (*Olea europaea* L.) plants, resulting in increased leaf dry weight, RWC, proline content, and photosynthesis [43]. Similarly, in bean (*Phaseolus vulgaris* L.), Se biofortification resulted in enhanced shoot and root fresh weight, chlorophyll, carotenoid, RWC, proline, total soluble sugars, peroxidase, and catalase enzymes when plants were exposed to a concentration of 50 mM NaCl [44]. Additionally, foliar Se application improved photosynthesis and water use efficiency (WUE) in tomato plants under salinity stress conditions, leading to increased tomato plant growth and a reduction in oxidative stress-induced damage [45].

Because of the increasing demand for food and the widespread occurrence of salinity-affected soils, research on plant responses to salinity stress has rapidly expanded in recent decades. Sustainable cultivation practices such as grafting plants onto resistant rootstocks and biofortification have been proposed as promising alternative. Despite the extensive research conducted on grafting of cucumber, little is known on the interactive effects of grafting and Se biofortification. Given the relative new subject of vegetable grafting in Iran, a study was designed with the aim to investigate the effect of Se grafted cucumber performance under salinity stress conditions.

## Materials and methods

### Plant materials and experimental treatments

In this study, the experimental treatments were implemented in a factorial design, based on completely randomized design with three replications. The research was conducted in a greenhouse at Razi University of Agriculture and Natural Resources. The first factor consisted of different salinity levels namely 0, 50, and 100 mM of sodium chloride; the second factor was the foliar application of Se at three levels of 0, 5, and 10 mg L^-1^ of sodium selenate, and the third factor included both grafted and non-grafted plants. Selenium foliar application was performed using sodium selenate salt and was applied simultaneously with the induction of salinity stress and Se foliar spraying was done once. Sodium selenate was purchased from Sigma Company.

**Fig. 1:**
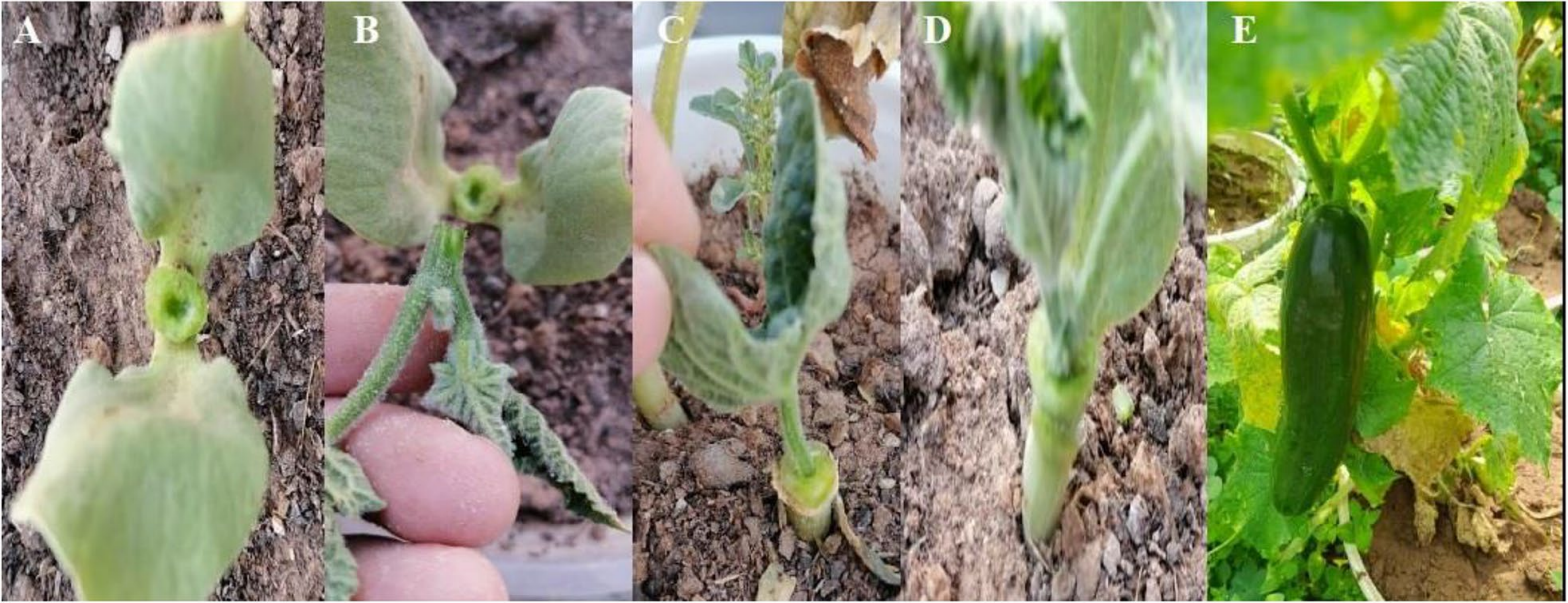
The Steps to perform the grafting, Preparation of the rootstock (**A**), Preparation of scion (**B**), Placing the scion on the rootstock (**C**), Fusion of the graft site (**D**) and Fruit formation on grafted cucumber (**E**)

Cucumber seeds of the Nagene variety and pumpkin seeds were obtained from the Pakan Bazr Isfahan Company. The seeds were sown in 5 cm diameter plastic containers with an equal mixture of soil, sand, and rotten manure. Pumpkin seeds were planted three to four days prior to cucumber seeds to ensure compatibility in the stem diameter between the rootstock and the scion to ensure success in grafting. After 25 days of seed cultivation, the Hole insertion grafting was performed as follows: the rootstock, which had cotyledon and true leaves, was prepared by carefully removing the true leaf and the rootstock terminal (apical) bud. A hole of 1-1.5 cm is made in the stem center using a toothpick. The scion plant, consisting only of cotyledons, was cut approximately 2 cm below the cotyledons. Finally, the scion was inserted into the hole created in the rootstock. The Steps to perform the grafting are shown in Fig. 1. Grafted plants were then transferred to 10 L plastic pots. Subsequently, the grafted seedlings were placed in grafting chambers with a relative humidity of 95%, complete darkness, and a temperature of 27–29 ºC. After three days, the relative humidity was steadily reduced until it reached the appropriate levels for optimum growth for the upcoming 14 days. Once the grafted plants had acclimatized and developed three true leaves, salinity stress was applied by adding NaCl to the irrigation water up to the end of the growth period. The salinity level increased gradually to reach the desired stress level. Selenium foliar spraying was done once and simultaneously with the application of salt stress. Pest and disease control, plant pruning and support for vertical growth, irrigation, and temperature and humidity regulation were all provided for grafted and non-grafted seedlings. The environmental conditions of the greenhouse during the cucumber growth period included a day temperature of 22–26 °C and the night temperatures of 18–20 °C, light intensity 6000–10,000 lx and relative humidity between 50 and 70%.

### Morphometric parameters

The morphological features studied were plant height, fresh weight of root, shoot and fruit, number of leaves and fruits, single plant yield, and total yield. Fruits were harvested three times per week between May 4 and June 20. At each harvest, the total number of fruits and weight of fruits was recorded separately. Mean fruit weight and total fruit yield were calculated.

### Physiologic parameters

#### Photosynthetic pigments

The amount of leaf chlorophyll was measured according to the method of Lichtenthaler [46]. Briefly, 0.1 g of fresh tissue of cucumber leaf was homogenized with 10 ml of 80% acetone inside the mortar. The obtained homogeneous mixture was centrifuged for 10 min at 3000 rpm, and finally, the upper part of the extract was separated. The absorbance of the samples was read at 645, 663 and 470 nm using the spectrophotometric method (Kerry 100 model, Varian, America), and the amount of chlorophyll and carotenoid was calculated in mg FW^-1^ using the following formulas:

$${\bf{Chla}}\, ({\rm{mgL}} - 1) = \left( {12.7 \times {\bf{A}}663} \right) - (2.69 \times {\bf{A}}645)$$

$${\bf{Chlb}}\, ({\rm{mgL}} - 1) = \left( {25.8 \times {\bf{A}}645} \right) - (4.68 \times {\bf{A}}663)$$

$${\bf{Chltotal}}\, ({\rm{mgL}} - 1) = \left( {20.21 \times {\bf{A}}645} \right) + (8.02 \times {\bf{A}}633)$$

$${\bf{Car}}\, ({\rm{mgL}} - 1) = \frac{{[(100 \times {\bf{A}}470 - 2.27 \times {\bf{Chla}} - 81.4{\bf{Chlb}}]}}{{227}}$$

#### RWC

For RWC assay, the fresh leaf discs (with 1.5 cm diameter) were weighted (FW), placed in a petri dish containing 30 ml cool distilled water for 24 h and then their turgid weight (TW) measured. To measure dry weight (DW), leaf discs were dried in an oven (35 L smart model, Shimaz Company, Iran) for 48 h at 72 ºC and then weighed. The leaf RWC was calculated as following formula [47]:

$${\bf{RWC}}\% = \frac{{({\bf{FW}} - {\bf{DW}})}}{{({\bf{TW}} - {\bf{DW}})}} \times 100$$

#### Electrolyte leakage (EL)

The method of Ben Hamed et al. (2007) was used to measure the leakage of electrolytes [48]. Firstly, the cucumber leaf segments (discs with 1.5 cm diameter) were immersed in 40 ml deionized water and the tubes shaken (120 rpm) immediately for 12 h under room temperature, and the electrical conductivity of the samples (EC_1_) was measured with an EC meter (Jenway model, England company). Then, they were autoclaved at 121 °C for 20 min, and after reaching 25 °C, the electrical conductivity of the samples (EC_2_) was measured again, and the ion leakage percentage was calculated from the following equation.

$${\bf{EL}} = \frac{{{\bf{EC}}1}}{{{\bf{EC}}2}} \times 100$$

#### Proline and soluble carbohydrates

The first 0.5 g of leaf tissue was ground with liquid nitrogen in a mortar. Then, 5 ml of 95% ethanol was immediately added to it and shaken vigorously. The upper part of the resulting solution was separated, and its sediments were washed twice with 5 ml of 70% ethanol, and their upper phase was added to the previously collected supernatant. The obtained solution was centrifuged at 3500 rpm for 10 min. After separating the liquid and solid phases, the liquid part was kept inside the refrigerator at a temperature of 4 ºC.

To determine the amount of proline, 1 ml of the above-mentioned alcoholic extract was diluted with 10 ml of distilled water, and 5 ml of Ninhydrin reagent was added to it. The composition of the Ninhydrin reagent for each sample included 0.125 g of Ninhydrin + 2 ml of 6 M Phosphoric acid and 3 ml of Glacial acetic acid. After adding the Ninhydrin reagent, 5 ml of Glacial acid was added, and the resulting mixture was placed in a boiling water bath at 100 ºC for 45 min. After removing the samples from the boiling water bath and cooling them, 10 ml of benzene was added to each sample and shaken vigorously until proline entered the benzene phase. The samples were then left to stand still for 30 min. Standard solutions of proline were prepared with concentrations of 0 to 0.1 µmol ml^-1^. Finally, the light absorption of standard solutions and samples was measured at a wavelength of 515 nm with a spectrophotometer (Kerry 100 model, Varian, America) [49].

To calculate the soluble sugars, 0.1 ml of the alcoholic extract was added to 3 ml of freshly prepared anthrone (150 mg of anthrone + 100 ml of 72% sulfuric acid). It was placed in a boiling water bath for 10 min. At this time, a colored substance was formed. Glucose standards were prepared from 0 to 0.1 µmol ml^-1^. Finally, the light absorption of standard solutions and samples was read with a spectrophotometer (Kerry 100 model, Varian, America) at a wavelength of 625 nm [50].

#### Total phenol and flavonoid content

To prepare methanolic extract, 0.5 g the fresh tissue of the leaf was crushed well in a mortar in the presence of 3 ml of 85% methanol and then smoothed. This methanolic extract was used to measure total phenol and flavonoids.

The amount of total phenol was determined using Folin–Ciocalteu reagent [51]. In this method, 300 µl of methanolic extract was mixed with 1500 µl of diluted folin solution (10:1 ratio with distilled water). After keeping it for 8 min at 25 °C, 1200 µl of 7% sodium bicarbonate solution were added. After 90 min of shaking on a shaker at a speed of 120 rpm at room temperature and in the dark, the absorbance of the samples was measured with a spectrophotometer at a wavelength of 765 nm (model Kerry 100, Varian, America). Using the standard curve of gallic acid, total phenol was calculated as mg of gallic acid g^-1^ FW.

The amount of total flavonoid was measured by the aluminum chloride calorimetric method [52]. Fifty µl of methanol extract was mixed with 10 µl of aluminum chloride (10%), 10 µl of potassium acetate (1 M), and 280 µl of deionized water. After vortexing, the samples were kept at room temperature for 40 min. The absorbance of the samples was read at a wavelength of 415 nm with a spectrophotometer. Total flavonoids were calculated in mg of quercetin g^-1^ FW using the quercetin standard curve.

#### Total protein content

The Bradford method [53] was employed to determine the amount of soluble proteins. For this purpose, 0.5 g of fresh leaves was mixed with 6.25 ml of extraction buffer solution and kept for 24 h. To prepare 1 L of extraction buffer solution, 121.4 g of Tris was dissolved in 1 L of distilled water, and the acidity of the solution was changed to 6.8 by normal hydrochloric acid until the desired buffer solution was obtained. After the mentioned period, the leaves were wholly ground in a mortar and then centrifuged at 6000 rpm for 20 min. Then, the sampler took 0.1 ml of the centrifuged upper solution, and 5 ml of Bio-Rad reagent was added to it. 100 mg of Coomassie Brilliant Blue − 250 was mixed with 50 ml of pure ethanol to prepare the reagent, and then it was brought to a volume of approximately 800 ml with distilled water and filtered. Finally, the volume of the filtered solution was increased to 1000 ml with 100 ml of pure phosphoric acid and distilled water. The resulting solution was placed in a spectrophotometer (Kerry 100 model, Varian, America) along with the extraction buffer solution, and its absorbance was read at a wavelength of 595 nm. To prepare the standard solution, 100 mg of bovine albumin was dissolved in 1 ml of extraction buffer and then made up to 1000 ml with distilled water. Then, a standard of 10 to 90 ppm was prepared from the solution, and its absorbance was read with a spectrophotometer at the mentioned wavelength.

#### Antioxidant enzyme activities

Enzyme extract was first prepared to measure the activity of antioxidant enzymes. Briefly, the frozen leaf tissue was first ground in a mortar in the presence of liquid nitrogen, and 0.1 g of it was added to a plastic tube containing 1 ml of extraction buffer and mixed. The sample was passed through a strainer, and the prepared extract was centrifuged for 15 min at a speed of 10,000 rpm at a temperature of 4 ºC and the clear supernatant solution was slowly separated; the resulting solution was used to measure the activity of each of the antioxidant enzymes as described below.

To determine the activity of the catalase enzyme, first, 50 µl of plant extract was mixed with 3 ml of extraction buffer containing 50 mM sodium phosphate (pH 7.8) and 2 mM ethylenediaminetetraacetic acid (EDTA). The reaction of catalase enzyme was started by adding 5 µl of 30% hydrogen peroxide to this mixture. The changes in optical absorption of the samples were recorded at a wavelength of 240 nm for 10 min. Each unit of catalase enzyme activity was considered the amount of enzyme that reduces 1 µl of hydrogen peroxide per minute. The amount of enzyme activity was expressed as units per mg of leaf protein. Each unit of CAT activity was considered as the 1.0 ml enzyme that reduces 1.0 µmol H_2_O_2_ min^-1^ [54].

Peroxidase enzyme activity was measured by spectrophotometry [55]. The first, 3 ml of extraction buffer (50 mM sodium phosphate (pH 7.8) and 2 mM ethylenediaminetetraacetic acid (EDTA) was poured into both control and sample cuvettes to start the peroxidase enzyme reaction. Five µl of 30% hydrogen peroxide and 5 µl of glycol were added to them. These two cuvettes were placed in the spectrophotometer, and the number read became 0. Then, 50 µl of plant extract were added to the sample cuvette, and the changes in light absorption of the samples at 465 nm wavelength, which indicates the degree of degradation and decrease in H_2_O_2_ concentration, were recorded every 10 s for 120 s. Each unit of peroxidase enzyme activity was considered as the amount of enzyme that reduces 1 µl of H_2_O_2_ ml^-1^ min^-1^.

#### Sodium and potassium concentration

After washing, the leaf samples were dried, placed inside the envelope, and placed in an oven at 72 ºC for 48 h. After drying, the samples were milled, then 0.2 g of the milled samples were poured into the test tube, and 2 ml of concentrated nitric acid was added to them and placed in a water bath at 60 ºC for 60 min. After 60 min, the temperature was increased to reach 100 ºC and kept at this temperature for 90 min. After cooling down the test tubes containing the samples to the laboratory temperature, 0.2 ml of 37% hydrogen peroxide was added to the samples, and the samples were left for 30 min to complete the reaction. After 30 min, the samples were filtered and their final volume was diluted to 25 ml by distilled water. This extract was used to measure sodium and potassium elements by the flame photometer (450G electronic flame photometer) [56].

### Statistical analyses

The experimental treatments were implemented factorial, based on completely randomized design with three replications containing two vines per each replicate. Data were analyzed with SAS (9.1) statistical software. Mean comparisons were performed with Duncan’s multiple range test at the 5% level of significance. All data were presented as mean ± standard deviation.

## Results

### Morphometric parameters

Salinity, Se, and grafting significantly affected the growth characteristics of cucumber. The highest plant height (345.100 cm), fresh plant weight (135.833 g), fresh root weight (47.933 g), number of nodes (61.777), number of fruit (59.700), fresh fruit weight (72.733 g), plant yield (4342.2 g) and total yield (614.60 g m^-2^) were observed in grafted cucumbers with a concentration of 10 mg L^-1^ of sodium selenate and 0 mM of NaCl (Table 1). Salinity, Se and grafting had no significant effect on plant and root dry weight. According to the obtained results, Se has improved the effects of salinity in transplanted plants so that in all three levels of salinity stress, the growth characteristics and yield of cucumber increased significantly with the increase of Se.

**Table 1:** Mean comparison of effect different levels of salt stress, Se and grafting on some morphological characteristics cucumber plant. Means ± SD (n = 3)

Salt stress (mM)	Sodium selenate (mgL^− 1^)	Grafting	Plant height (cm)	Fresh plant weight (g)	Fresh root weight (g)	Number of nodes (-)	Number of fruits (-)	Fresh fruit weight (g)	Plant yield (g plant^− 1^)	Total yield (g m-^2^)
		Non-Grafting	276.06 ± 3.05^d^	97.933 ± 2.30^f^	32.170 ± 0.24^d^	40.583 ± 0.19^de^	46.413 ± 0.42^ef^	48.153 ± 0.43^de^	2235.1 ± 40.76^e^	316.36 ± 5.77^e^
	0	Grafting	301.05 ± 14.26^c^	106.225 ± 4.27^e^	32.995 ± 0.20^d^	40.583 ± 0.00^d^	48.025 ± 0.57^e^	50.340 ± 0.88^cd^	2418.7 ± 70.68^e^	342.35 ± 10.00^e^
		Non-Grafting	318.150 ± 2.48^b^	112.800 ± 5.19^d^	36.430 ± 2.88^c^	43.775 ± 1.66^c^	54.000 ± 1.61^d^	53.800 ± 1.73^c^	2735.1 ± 174.96^d^	387.14 ± 24.76^d^
0	5	Grafting	320.767 ± 0.40^b^	121.300 ± 3.46^c^	39.197 ± 0.23^b^	46.253 ± 0.89^c^	54.000 ± 1.55^c^	58.033 ± 2.36^b^	3136.3 ± 216.16^c^	443.92 ± 30.59^c^
		Non-Grafting	324.433 ± 0.75^b^	127.833 ± 5.60^b^	40.743 ± 2.37^b^	51.110 ± 1.92^b^	56.967 ± 2.36^b^	60.813 ± 0.85^b^	3465.7 ± 193.79^b^	490.54 ± 27.43^b^
	10	Grafting	345.100 ± 17.14^a^	135.833 ± 1.32^a^	47.933 ± 0.83^a^	61.777 ± 7.31^a^	59.700 ± 8.70^a^	72.733 ± 9.46^a^	4342.2 ± 565.27^a^	614.60 ± 80.01^a^
		Non-Grafting	200.687 ± 0.61^h^	76.467 ± 0.46^ij^	19.233 ± 0.40^h^	37.330d ± 0.00^ef^	29.660 ± 0.00^i^	43.627 ± 0.21^efgh^	1294.0 ± 6.50^i^	183.15 ± 0.92^i^
	0	Grafting	203.133 ± 1.50^h^	79.533 ± 2.19^hi^	23.680 ± 3.44^g^	37.330 ± 0.00^def^	30.107 ± 0.38^i^	44.893 ± 0.87^efg^	1351.8 ± 43.59^hi^	191.34 ± 6.16^hi^
50		Non-Grafting	220.553 ± 4.42^g^	83.200 ± 3.29^h^	27.063 ± 1.44^f^	37.587 ± 0.06^def^	34.997 ± 1.15^h^	46.000 ± 0.51^defg^	1610.2 ± 71.64^gh^	227.92 ± 10.14^gh^
	5	Grafting	229.220 ± 3.08^g^	87.933 ± 0.80^g^	29.223 ± 0.42^e^	38.320 ± 0.57^de^	36.577 ± 0.21^h^	46.667 ± 0.05^defg^	1706.9 ± 12.07^g^	241.60 ± 1.70^g^
		Non-Grafting	242.600 ± 5.19^f^	90.467 ± 3.05^g^	31.370 ± 0.19^de^	38.933 ± 0.05^de^	39.687 ± 4.08^g^	47.267 ± 0.28^def^	1876.6 ± 205.07^fg^	265.63 ± 29.02^fg^
	10	Grafting	256.400 ± 6.75^e^	95.467 ± 1.27^f^	18.533 ± 0.19^h^	39.220 ± 0.19^de^	45.133 ± 0.63^f^	47.600 ± 0.00^de^	2148.3 ± 30.23^ef^	304.08 ± 4.27^ef^
		Non-Grafting	116.333 ± 2.30^k^	47.933 ± 3.52^m^	12.530 ± 0.91^k^	27.033 ± 0.83^h^	18.993 ± 0.57^l^	36.033 ± 0.23^i^	684.5 ± 25.26^k^	96.88 ± 3.57^k^
	0	Grafting	131.667 ± 10.96^j^	55.333 ± 2.88^l^	13.863 ± 0.23^kj^	28.960 ± 0.83^h^	20.107 ± 0.38^l^	36.700 ± 0.34^i^	738.0 ± 21.08^k^	104.46 ± 2.98^k^
		Non-Grafting	141.667 ± 1.15^j^	61.267 ± 0.98^k^	14.733 ± 0.92^kj^	32.697 ± 0.06^g^	23.663 ± 2.30^k^	39.420 ± 0.15^hi^	933.0 ± 94.93^jk^	132.07 ± 13.43^jk^
100	5	Grafting	157.200 ± 12.29^i^	63.800 ± 1.21^k^	15.933 ± 0.11^ij^	32.923 ± 0.13^g^	26.997 ± 0.57^j^	40.333 ± 0.63^hi^	1089.1 ± 40.22^ij^	154.16 ± 5.69^ij^
		Non-Grafting	192.433 ± 0.75^h^	72.933 ± 1.44^j^	17.333 ± 0.57^hi^	34.553 ± 0.95^fg^	29.660 ± 0.96^ij^	42.233 ± 0.50^gh^	1191.7 ± 42.38^ij^	168.68 ± 5.99^ij^
	10	Grafting	196.833 ± 3.05^h^	75.267 ± 0.57^ij^	18.533 ± 0.46^h^	36.553 ± 0.77^ef^	29.660 ± 0.00^ij^	42.967 ± 0.57^fgh^	1260.2 ± 16.93^i^	178.37 ± 2.39^i^

Means indicated with similar letters in columns do not differ significantly at the 5% level

### Physiological characteristics

#### Physiological traits

The results showed that salinity and Se significantly affected the amount of photosynthetic pigments in cucumber leaf. Unlike Se, salinity harms the amount of photosynthetic pigments in cucumber leaf. The highest amount of chlorophyll a (4.966 mg g^-1^ FW), chlorophyll b (1.936 mg g^-1^ FW), total chlorophyll (6.933 mg g^-1^ FW), and carotenoid (0.596 mg g^-1^ FW) was observed in the treatment of 0 mM of NaCl along with 10 mg L^-1^ of sodium selenate. With increasing Se concentration, the amount of photosynthetic pigments in cucumber leaf increased in all three levels of salinity stress compared to the control. At the same time, with the increase of Se concentration in non-salinity conditions, the number of photosynthetic pigments increased (Table 2).

**Table 2:** Mean comparison of effect different levels of salt stress and Se on photosynthetic pigments cucumber leaf. Means ± SD (n = 3)

Salt stress (mM)	Sodium selenate (mg L^− 1^)	Chlorophyll a (mg g^− 1^ FW)	Chlorophyll b (mg g^− 1^ FW)	Total chlorophyll (mg g^− 1^ FW)	Carotenoid (mg g^− 1^ FW)
	0	4.266 ± 0.13^c^	1.300 ± 0.15^c^	6.300 ± 0.08^c^	0.550 ± 0.008^c^
0	5	4.666 ± 0.13^b^	1.600 ± 0.08^b^	6.633 ± 0.13^b^	0.576 ± 0.005^b^
	10	4.966 ± 0.05^a^	1.936 ± 0.10^a^	6.933 ± 0.05^a^	0.596 ± 0.005^a^
	0	2.966 ± 0.18^f^	0.616 ± 0.04^f^	5.033 ± 0.13^f^	0.486 ± 0.005^f^
50	5	3.500 ± 0.08^e^	0.936 ± 0.01^e^	5.600 ± 0.17^e^	0.503 ± 0.005^e^
	10	3.900 ± 0.08^d^	1.070 ± 0.01^d^	6.000 ± 0.08^d^	0.526 ± 0.005^d^
	0	1.423 ± 0.14^i^	0.286 ± 0.05^h^	1.773 ± 0.29^i^	0.406 ± 0.005^i^
100	5	2.100 ± 0.08^h^	0.413 ± 0.03^g^	4.033 ± 0.13^h^	0.430 ± 0.008^h^
	10	2.500 ± 0.17^g^	0.506 ± 0.02^g^	4.633 ± 0.13^g^	0.463 ± 0.01^g^

Means indicated with similar letters in columns do not differ significantly at the 5% level

#### Proline

According to the results (Table 3), with increasing levels of salinity stress and Se, the proline content of cucumber leaf increased compared to the control. The highest content of proline (32.667 mg g^-1^ FW) was found in grafted cucumber and 100 mM of NaCl along with 10 mg L^-1^ of sodium selenate.

**Table 3:** Mean comparison of effect different levels of salt stress, Se and grafting on some physiological characteristics cucumber leaf. Means ± SD (n = 3)

Salt stress (mM)	Sodium selenate (mg L^− 1^)	Grafting	Proline (mg g^− 1^ FW)	Soluble sugars (mg g^− 1^ FW)	Total Protein (mg g^− 1^ FW)	Total phenol (mg g^− 1^ FW)	Flavonoid (mg g^− 1^ FW)	Catalase (unit mg^− 1^ protein)	Peroxidase (unit mg^− 1^ protein)
		Non-Grafting	6.687 ± 0.00^g^	5.533 ± 0.23^p^	5.666 ± 0.17^o^	0.233 ± 0.01^m^	2.170 ± 0.00^m^	32.000 ± 0.46^m^	43.000 ± 0.57^m^
	0	Grafting	7.080 ± 0.49^g^	5.866 ± 0.05^op^	6.466 ± 0.11^n^	0.260 ± 0.01^lm^	2.390 ± 0.05^l^	32.666 ± 0.69^m^	45.667 ± 1.15^lm^
		Non-Grafting	7.373 ± 0.00^g^	6.100 ± 0.16^no^	7.133 ± 0.23^m^	0.296 ± 0.01^klm^	2.480 ± 0.04^k^	35.166 ± 0.17^l^	49.333 ± 0.57^kl^
0	5	Grafting	8.113 ± 1.15^g^	6.366 ± 0.10^mn^	7.800 ± 0.57^l^	0.343 ± 0.02^jkl^	2.533 ± 0.01^k^	36.166 ± 0.40^k^	53.333 ± 0.57^k^
		Non-Grafting	12.167 ± 5.19^f^	6.733 ± 0.75^lm^	8.266 ± 0.57^kl^	0.376 ± 0.04^ijk^	2.703 ± 0.005^j^	37.133 ± 0.17^j^	64.333 ± 10.96^j^
	10	Grafting	12.967 ± 7.50^f^	7.000 ± 0.57^lm^	8.800 ± 0.57^k^	0396 ± 0.19^ij^	2.796 ± 0.05^i^	37.800 ± 0.40^j^	68.333 ± 3.45^ij^
		Non-Grafting	13.800 ± 0.17^ef^	7.433 ± 0.05^jk^	9.800 ± 0.17^j^	0.450 ± 0.01^hi^	2.900 ± 0.03^h^	39.333 ± 0.57^i^	73.333 ± 0.57^hi^
	0	Grafting	14.400 ± 0.34^ef^	7.700 ± 0.03^ij^	10.333 ± 0.28^ij^	0.483 ± 0.01^h^	2.980 ± 0.03^h^	40.333 ± 0.28^h^	75.333 ± 1.15^gh^
50		Non-Grafting	15.167 ± 0.28^def^	8.066 ± 0.01^hi^	10.800 ± 0.17^hi^	0.503 ± 0.005^gh^	3.126 ± 0.01^g^	41.666 ± ^1.15^	77.333 ± 0.57^fgh^
	5	Grafting	17.167 ± 1.44^cde^	8.400 ± 0.04^h^	11.200 ± 0.17^h^	0.516 ± 0.005^gh^	3.186 ± 0.04^g^	43.466 ± 0.40^f^	79.333 ± 1.15^fg^
		Non-Grafting	18.733 ± 0.23^cd^	9.633 ± 0.07^g^	12.233 ± 0.40^g^	0.586 ± 0.01^fg^	3.283 ± 0.07^f^	45.066 ± 0.11^e^	82.333 ± 2.30^ef^
	10	Grafting	19.000 ± 0.00^cde^	9.900 ± 0.04^fg^	12.900 ± 0.17^f^	0.626 ± 0.02^fe^	3.416 ± 0.04^e^	45.200 ± 8.70^e^	85.667 ± 0.57^de^
		Non-Grafting	19.000 ± 0.23^cd^	10.333 ± 0.23^ef^	13.600 ± 0.28^e^	0.660 ± 0.005^def^	3.470 ± 0.08^de^	47.266 ± 0.00^d^	90.333 ± 1.73^cd^
	0	Grafting	19.650 ± 0.10^c^	10.750 ± 0.05^de^	14.100 ± 0.40^e^	0.700 ± 0.01^cde^	3.542 ± 0.10^cd^	48.700 ± 0.57^c^	92.750 ± 0.57^c^
		Non-Grafting	20.000 ± 0.12^c^	11.140 ± 0.17^cd^	14.800 ± 0.23^d^	0.730 ± 0.02^cd^	3.620 ± 0.01^bc^	49.150 ± 0.28^bc^	94.500 ± 0.57^c^
100	5	Grafting	21.333 ± 0.51^c^	11.406 ± 0.05^c^	15.666 ± 0.34^c^	0.766 ± 0.01^cb^	3.673 ± 0.02^b^	49.766 ± 0.57^b^	95.667 ± 2.88^c^
		Non-Grafting	26.000 ± 0.28^b^	12.126 ± 0.05^b^	17.333 ± 0.11^b^	0.823 ± 0.01^b^	3.893 ± 0.05^a^	50.800 ± 0.23^a^	103.333 ± 0.57^b^
	10	Grafting	32.667 ± 0.40^a^	13.666 ± 0.17^a^	18.666 ± 0.34^a^	1.090 ± 0.005^a^	3.966 ± 0.02^a^	51.466 ± 0.34^a^	120.000 ± 2.88^a^

Means indicated with similar letters in columns do not differ significantly at the 5% level

#### Soluble carbohydrates

The amount of soluble carbohydrates increases as salinity and Se levels increased. Only in the highest salinity stress and Se level did the results (Table 3) show a significant difference between grafted and non-grafted cucumber plants. The highest amount of soluble carbohydrates (13.666 mg g^-1^ FW) was observed in grafted cucumber and 100 mM of NaCl along with 10 mg L^-1^ of sodium selenate.

#### Total protein

In grafted and non-grafted cucumber, with the increase of Se and salinity level, the amount of total protein increased, which was significantly different from the control treatment. Thus, the highest amount of total protein (18.666 mg g^-1^ FW) was observed in grafted cucumber with 100 mM of NaCl along with 10 mg L^-1^ of sodium selenate.

#### Total phenol and flavonoid

With increasing Se concentration, the total phenol content of cucumber leaf increased under salinity stress condition. The highest amount of total phenol (1.090 mg g^-1^ FW) and total flavonoid (3.966 mg g^-1^ FW) were in the treatment of 100 mM of NaCl and 10 mg L^-1^ of sodium selenate of grafted plants, significantly different from the control treatment (Table 3).

#### Antioxidant enzymes

With increasing Se concentration and salinity stress, catalase and peroxidase enzymes activity increased (Table 3). The results show no significant difference between grafted and non-grafted plants at the lowest and highest concentrations of Se and salinity stress. Still, grafted cucumbers had higher catalase and peroxidase enzyme activity under salinity stress conditions than non-grafted plants (Table 3).

#### RWC

**Fig. 2:**
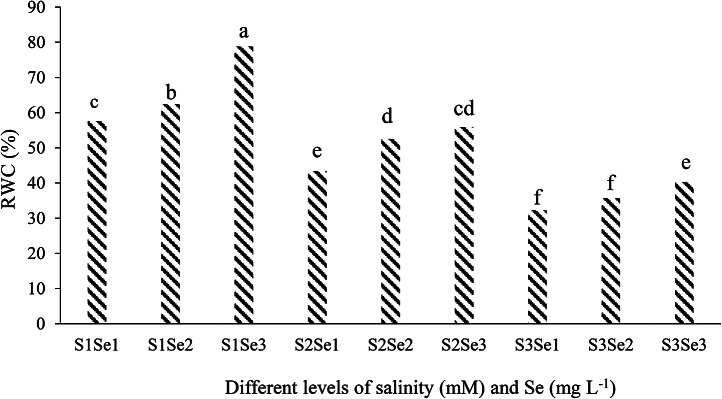
Mean comparison of effect different levels of salinity and Se on RWC cucumber leaf. S_1_, S_2_ and S_3_ respectively: 0, 50, and 100 mM of NaCl and Se_1_, Se_2_ and Se_3_ respectively: 0, 5, and 10 mg L^-1^ sodium selenate

According to the results of mean comparison of effect different levels of salinity and Se on RWC cucumber leaf (Fig. 2), unlike salinity, Se increased the amount of RWC. The highest amount of RWC (78.77%) was in the treatment of 0 mM of NaCl and 10 mg L^-1^ of sodium selenate (Fig. 2). According to the obtained results, in the condition of salt stress, with the increase of Se level, the amount of RWC increased.

#### EL

**Fig. 3:**
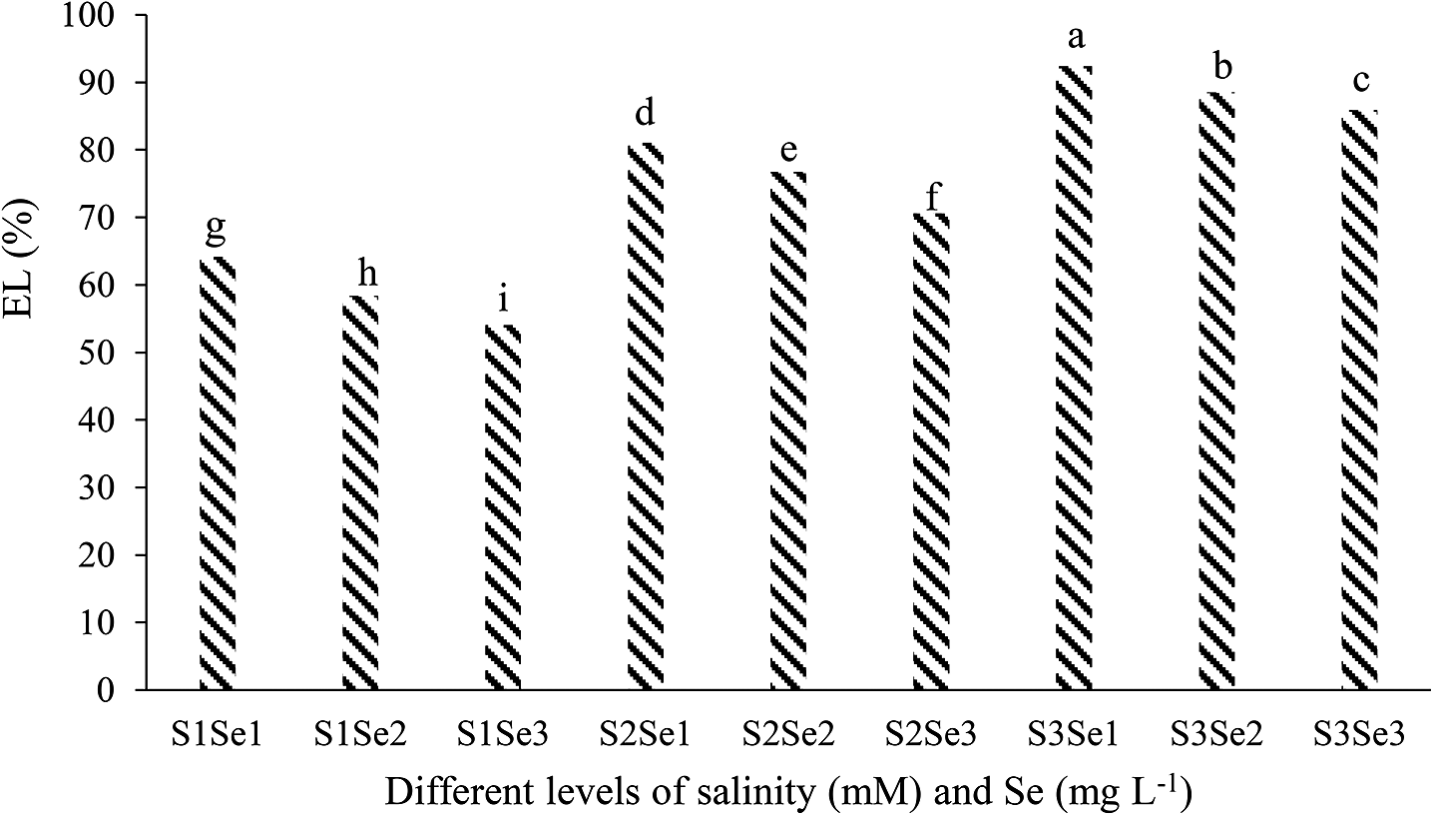
Mean comparison of effect different levels of salinity and Se on EL cucumber leaf. S_1_, S_2_ and S_3_ respectively: 0, 50, and 100 mM of NaCl and Se_1_, Se_2_ and Se_3_ respectively: 0, 5, and 10 mg L^-1^ sodium selenate

The results mean comparison of effect different levels of salinity and Se on EL cucumber leaf show that, contrary to Se, salinity has increased the amount of EL in cucumber leaf. The maximum amount of EL of cucumber leaf (92.37%) was in treating 100 mM of NaCl and 0 mg L^-1^ of sodium selenate (Fig. 3).

#### Sodium and potassium concentration

The amount of potassium decreased as salinity stress increased, but the amount of sodium increased. Selenium reduced the quantity of sodium while increasing the amount of potassium in cucumber leaf and root (Table 4). The highest amount of potassium in leaf (20.566 mg g^-1^ DW) and root (32.523 mg g^-1^ DW) of cucumber was in treating 0 mM NaCl with 10 mg L^-1^ sodium selenate. In contrast, the highest amount of sodium was observed in treating 100 mM of NaCl and 0 mg L^-1^ of sodium selenate (Table 4).

**Table 4:** Mean comparison of effect different levels of salt stress and Se on sodium and potassium concentration cucumber root and leaf. Means ± SD (n = 3)

Salt stress (mM)	Sodium selenate (mg L^− 1^)	Leaf potassium (mg g^− 1^ DW)	Root potassium (mg g^− 1^ DW)	Leaf sodium (mg g^− 1^ DW)	Root sodium (mg g^− 1^ DW)
	0	16.796 ± 0.24^c^	20.250 ± 2.18^c^	1.260 ± 0.05^ef^	25.616 ± 1.18^g^
0	5	18.210 ± 0.42^b^	28.510 ± 0.83^b^	1.116 ± 0.14^fg^	20.126 ± 0.33^h^
	10	20.566 ± 0.89^a^	32.523 ± 2.22^a^	0.930 ± 0.52^g^	17.700 ± 0.80^i^
	0	15.343 ± 0.23^e^	16.173 ± 0.87^e^	2.036 ± 0.05^c^	36.363 ± 0.41^d^
50	5	15.850 ± 0.17^de^	17.086 ± 0.18^de^	1.713 ± 0.22^d^	30.973 ± 1.49^e^
	10	16.313 ± 0.16^cd^	17.793 ± 0.50^d^	1.436 ± 0.04^e^	28.726 ± 1.94^f^
	0	12.093 ± 0.71^h^	8.260 ± 0.47^h^	3.140 ± 0.11^a^	44.796 ± 0.45^a^
100	5	13.113 ± 0.37^g^	9.766 ± 0.34^g^	2.486 ± 0.02^b^	43.260 ± 0.36^b^
	10	14.643 ± 0.43^f^	13.393 ± 1.31^f^	2.186 ± 0.03^c^	41.180 ± 1.03^c^

Means indicated with similar letters in columns do not differ significantly at the 5% level

## Discussion

Salinity seriously threatens agricultural and horticultural crops, leading to reduced growth and yield. Growth is directly related to plant productivity and yield. As a result, it has been widely recognized as a critical indicator in most physiological research. Salinity decreases turgor pressure and DNA synthesis by inducing osmotic stress, ion imbalance, and ion toxicity [45]. All salinity-induced changes in plant metabolism, which include physiological and biochemical processes such as photosynthesis, ion homeostasis, and antioxidant activity, lead to reduced growth [57].

Salt stress has reduced the growth characteristics of grafted and non-grafted cucumbers. In this study, the height and fresh weight of cucumber plant and fruit decreased under salt stress compared to the control (Table 1). The weight loss of air parts under salinity stress conditions can be due to the accumulation of harmful ions such as chlorine and sodium, which are detrimental or cause disturbances in the absorption of water and other minerals. Also, salinity increases the amount of energy required to maintain the cell’s standard conditions, and as a result, less energy is left for growth needs [58]. Under salinity stress conditions, the absorption and transfer of water and minerals from the roots to the leaves decrease. The plant reduces its photosynthetic level by reducing the number and surface of the leaves, which also reduces the plant’s photosynthetic capacity [59]. According to the results of this research, salinity stress, by lowering RWC leaf and causing ion toxicity, led to a decrease in the growth characteristics, yield and yield components of cucumber, which is consistent with the results of Semida et al. (2021) in onion (*Allium cepa*) and Karaca et al. (2023) in tomato [60, 61].

Similar to the outcomes shown in cucumber [62] and garlic (*Allium sativum* L.) [32], under salinity stress conditions, foliar application of Se (10 mg L^-1^ sodium selenate) improved vegetative traits compared to the control treatment. Transferring them to the roots through water and nutrient elements absorption, the osmotic balance is essential in plant tolerance to salinity stress conditions, which is associated with increasing plant growth characteristics [63]. Under salinity stress conditions, Se causes an increase in the RWC, which leads to more water retention in the tissues. Under salinity stress, Se can be said to increase the absorption of macronutrients like magnesium, potassium, phosphorus, and nitrogen while decreasing the absorption of sodium. It also increases proline levels, RWC, and the activity of antioxidant enzymes [64], thereby resulting in enhanced cucumber yield (Table 1).

Cucumber is sensitive to salinity; thus, identifying salinity-tolerant rootstock appears to be critical. Pumpkin has shown more tolerance to environmental stress than cucumber [65]. According to the results (Table 1), no significant difference was observed between grafted and non-grafted cucumbers in the treatment without salinity stress. Still, at high levels of salinity stress, the growth characteristics studied in grafted plants were higher than non-grafted plants; this shows the positive effect of grafting and using pumpkin rootstock under salinity stress conditions and follows the results obtained in the pepper (*Capsicum annuum* L.) [66]. According to the conducted studies, grafting can affect water absorption and nutrient elements [67, 68]. In the present study, grafting and foliar application of Se improved the growth characteristics of cucumber under salinity stress conditions. In salinity stress conditions, grafting tomato on eggplant (*Solanum melongena* L.) has improved tomato fruit’s physiological condition and yield. Eggplant as a rootstock increases the amount of proline and antioxidant enzymes, including catalase, and decreases the amount of sodium in grafted plants [69], according to what was observed in our research. Grafting tomato on potato under salinity stress conditions increased the yield of tomato fruit. The interaction between the rootstock and scion can increase the scion’s growth and biomass and also play a role in the distribution of assimilates between the source (leaf, stem, and root) and the sink (fruit) [18]. Rootstock will play an important role in fruit yield and growth, and there are many reports on increasing tolerance to environmental stress in grafted fruit trees. At the same time, pumpkin rootstock and Se were tested for the first time for cucumber salinity tolerance increase (Table 1). Pumpkin rootstock and Se significantly increased plant height, number of leaves, and fruit yield of grafted cucumber, which can be attributed to the increase of compatible osmolytes (proline, total soluble sugars), antioxidant enzymes, and reduction of sodium absorption and transport under salinity stress conditions. Some studies employed foliar Se application and grafting to increase tomato fruit growth, yield, and quality. According to the results, grafting with 2 and 4 µg L^-1^ of Se resulted in increased cherry tomato yield and nutritional compounds [70].

In salinity stress conditions, Se has increased the amount of photosynthetic pigments, consistent with the results of Hawrylak-Nowak (2009) in cucumber plants [71]. In garlic, by applying 8 mg L^-1^ of sodium selenate under salinity stress conditions the amount of chlorophyll and carotenoid was increased [32]. The reduction of photosynthetic pigments under salinity stress conditions has been reported in various plants [72], which can be due to the accumulation of sodium ions in chloroplasts, the deterioration of chloroplast and thylakoid membranes, the reduction of enzymes responsible for the synthesis of photosynthetic pigments, the reduction of the stability of pigment-protein complexes due to the presence of ions, the prevention of new chlorophyll biosynthesis due to the synthesis of more proline, the lack of magnesium and potassium ions - as the main elements in the synthesis of chlorophyll, the reduction of the ratio of potassium to sodium, the attack of ROS caused by oxidative stress and peroxidation, the decomposition of chlorophyll and the activation of the chlorophyllase enzyme, and finally the reduction of the content of chlorophyll [73]. The foliar application of Se can increase the content of chlorophyll and carotenoids in the leaves of plants subjected to salinity stress conditions by reducing oxidative tension and preventing the destruction of chlorophyll molecules. Carotenoids have a protective role against oxidative stress and are also effective in detoxifying chlorophyll and reducing the toxic effects of free radicals [74].

Carbohydrates, under stress conditions, in addition to playing a role in osmoregulation, also have a protective role against oxidative stress through free electrons in their structural rings [75]. It appears that using Se to increase soluble carbohydrates production is a viable step in plant protection against oxidative stress. Basil (*Ocimum basilicum* L.) has also shown a rise in soluble carbohydrates under salinity stress conditions [76].

The findings showed that grafting positively affected the amount of proline in cucumber leaves under salinity stress conditions so that the highest amount of proline was in the grafted cucumber and the treatment of 0 mM of NaCl with 10 mg L^-1^ of sodium selenate. These findings were consistent with those obtained in tomato [77] and pepper [66]. Proline is one of the compatible substances most plants produce under stress conditions and helps maintain osmotic balance. In fact, the increase of proline in plants under salinity stress is the plant’s reaction to reducing water potential in the root environment [78]. By lowering the osmotic potential of root cells, proline creates water and nutrient absorption conditions. At the same time, proline induces the transcription of salinity stress-resistant proteins so that the plant tolerates salinity stress conditions [79]. Raising the proline level with foliar Se application may help plants resist salinity stress by enhancing the antioxidant defense system. Under salinity stress conditions, Se leads to increased accumulation of some compatible osmolytes, including proline and total soluble sugars [80]. According to our results, Se increased the amount of chlorophyll a and b, proline, and catalase enzyme activity in salinity stress in garlic plant [32].

According to the findings (Table 3), salinity and Se positively affected total soluble protein in grafted cucumber leaf compared to control plants. The highest amount of cucumber leaf protein was observed in the treatment of grafted plants with 100 mM of NaCl and 10 mg L^-1^ of sodium selenate (Table 3). Selenium enhances the amount of proteins under salinity stress conditions by protecting proteins with sulfhydryl groups and stimulating the nitrate reductase enzyme gene transcription [81]. Under salinity stress conditions, Se enhanced the amount of protein and soluble carbohydrates in wheat (*Triticum aestivum* L.), according to the studies [41].

According to the results of comparing the averages (Table 3), in high levels of salinity stress and Se, no significant difference in the flavonoid content of cucumber leaf was seen between grafted and non-grafted plants. While with increasing Se concentration and salinity stress, the flavonoid content of cucumber leaf increased (Table 3). Phenolic compounds are essential in inhibiting lipid peroxidation and scavenging free radicals. In salinity stress conditions, the increase of phenolic compounds is directly related to the production of free radicals. The rise in phenolic compounds under salinity stress conditions is a plant stress-resistance mechanism [82]. At the same time, the increase of phenolic compounds under salinity stress conditions is associated with an increase in the production of lignins, which helps to increase the plant’s resistance to stresses [83]. Under salinity stress conditions, Se can play a protective role by activating the production of the phenylalanine ammonia-lyase enzyme, which is necessary for producing phenolic compounds, and lead to an increase in phenolic compounds [84]. According to the results, Se has increased proline, total phenol, flavonoid, and antioxidant enzymes in stevia (*Stevia rebaudiana* Bertoni) under salinity stress [85]. Flavonoids are essential because of their role in non-enzymatic defense systems. The amount of flavonoids is significantly affected by environmental conditions. When a plant detects stress, its defensive mechanism, which includes flavonoids, is activated and strengthened to deal with the stress [78].

Selenium and salinity stress positively affected peroxidase enzyme activity (Table 3). The maximum amount of peroxidase enzyme activity was detected in the grafted plants with the highest Se concentration and salinity stress. That exhibited a significant difference from the control treatment. The antioxidant system helps protect cells against free radicals. In this study, the activity of antioxidant enzymes such as catalase and peroxidase increased under salinity stress conditions and foliar application of Se (Table 3), following the results obtained in the Stachys byzantina (*Stachys byzantine* L.) [86]. Environmental stresses are associated with oxidative stress and increased production of ROS, which destroy membranes and peroxidation of lipids. ultimately causing the leakage of materials the cell and cell death. Plants can clear these free radicals against oxidative stress using their antioxidant system, which includes ascorbate peroxidase, catalase, superoxide dismutase, peroxidase, and non-enzymatic antioxidants like ascorbate, glutathione, and alphatocopherol, according to the findings of this study [78]. By increasing the activity of antioxidant enzymes, Se causes the removal of active oxygen and, as a result, reduces the oxidation of lipids in membranes and the surface of malondialdehyde [87]. Selenium suppresses the enzymes glutathione peroxidase and hydrogen peroxide, then the enzymes ascorbate peroxidase, catalase, and glutathione reductase clean up the hydrogen peroxide residues [88].

The highest RWC of cucumber leaf was seen in the highest concentration of Se (10 mg L^-1^ sodium selenate) in all three degrees of salinity stress. Selenium increased the RWC of cucumber leaf under salinity stress conditions (Fig. 2). Measuring the RWC is one indicator that estimates the plant’s resistance to salinity stress. Salinity lowers the cultivation bed’s water potential, affects the volume of water the cucumber roots can absorb, and ultimately lowers the RWC. Selenium helps to maintain the osmotic pressure of cucumber in salinity stress by producing compatible osmolytes. The production of compatible osmolytes reduces the osmotic pressure inside the cell, which helps keep the water inside and prevents the cell from drying out. By helping to absorb water from the soil solution, it increases the water pressure and the relative amount of RWC [89].

In all three concentrations of salinity stress, with increasing Se concentration, the amount of electrolyte leakage of cucumber leaf has decreased (Fig. 3). In salinity stress, electrolyte leakage decreases as antioxidant activity increases, and Se reduces electrolyte leakage by protecting cell membranes [90]. Because the cell membrane is a primary target in many environmental stresses, including salinity, membrane stability under stress conditions is one of the indicators of tolerance [91]. Therefore, measuring the amount of electrolyte leakage is one of the good indicators for measuring the amount of oxidative damage to the membrane. Due to the cytoplasmic membrane’s vulnerability, the cell’s contents leak out, and the amount of this damage is determined by measuring electrolyte leakage [92].

The fundamental strategy for regulating solute accumulation in the plant is reducing sodium transport from the root to the air organ and absorbing more potassium than sodium. Salinity lowers potassium levels in cucumber plant root (Table 4); indeed, one of the harmful impacts of salinity is the disruption of potassium absorption. The reduction of potassium absorption due to the presence of the competing sodium ion is due to the similarity of the size of the hydrated radius of these two ions [62]. As a result, their transfer proteins are misdiagnosed. Because of the presence of high levels of sodium in the surrounding environment of the roots during salinity stress, in addition to disrupting potassium absorption and causing damage to the root membranes, the selective selection of these membranes also changes [93]. Potassium is one of the most abundant elements in plants, and it is required to form proteins, enzymes, and photosynthesis. It plays a role in regulating osmotic potential, and with increasing pH and sodium, its availability for plants decreases [91]. Under condition of salinity stress, Se increases potassium. It decreases the amount of sodium in the seedling index by binding sodium to the root cell wall, reducing salinity stress damage. Sodium ion blocks potassium ion transport channels on the membrane surface, while Se can affect gene expression of sodium transporters and hydrogen pumps. The appropriate Se concentration can increase the expression of tonoplasty H^+^ ATPase and Na^+^/H^+^ antiport in the root membrane, reducing sodium ion transport to the restricted aerial parts and toxicity [87]. According to the findings (Table 4), the root contains higher sodium than the cucumber leaf that, according to the observations of Gou et al. [64]., in grafted cucumber on rootstock pumpkin lowers sodium transfers from root to leaf (Table 4).

## Conclusions

Considering that for the first time the effect of selenium and transplantation on salinity tolerance of greenhouse cucumber was studied, the results showed that pumpkin has more tolerance to salt stress than cucumber, which is accompanied by increase in growth characteristics, compatible osmolytes (proline, total soluble sugars), compounds (total phenol, flavonoid), antioxidant enzymes (catalase) and accumulation potassium of cucumber leaf. Selenium, along with grafting, had an effective role in increasing salt tolerance in cucumber. As a result, it can be said that the use of pumpkin rootstock and 10 mg L^-1^ of sodium selenate is a good strategy to tolerate salinity and improve the quality and yield of grafting cucumber plants.

## Data Availability

All data are available in the manuscript file.
